# ICHD-3 is significantly more specific than ICHD-3 beta for diagnosis of migraine with aura and with typical aura

**DOI:** 10.1186/s10194-019-1072-2

**Published:** 2020-01-07

**Authors:** Carl H. Göbel, Sarah C. Karstedt, Thomas F. Münte, Hartmut Göbel, Sebastian Wolfrum, Elena R. Lebedeva, Jes Olesen, Georg Royl

**Affiliations:** 1 Department of Neurology, University of Lübeck, 23538 Lübeck, Germany; 2 Institute of Psychology II, University of Lübeck, 23538 Lübeck, Germany; 3 Kiel Migraine and Headache Centre, 24149 Kiel, Germany; 4 Interdisciplinary Emergency Department, University of Lübeck, 23538 Lübeck, Germany; 5 Department of Neurology, Ural State Medical University, Yekaterinburg, Russia; 6 Danish Headache Center, Department of Neurology, Glostrup Hospital, University of Copenhagen, Copenhagen, Denmark

**Keywords:** Migraine with Aura, Transient ischemic attack, ICHD-3, Emergency room

## Abstract

**Background:**

In the emergency room, distinguishing between a migraine with aura and a transient ischemic attack (TIA) is often not straightforward and mistakes can be harmful to both the patient and to society. To account for this difficulty, the third edition of the International Classification of Headache disorders (ICHD-3) changed the diagnostic criteria of migraine with aura.

**Methods:**

One hundred twenty-eight patients referred to the emergency room at the University Hospital of Lübeck, Germany with a suspected TIA were prospectively interviewed about their symptoms leading to admission shortly after initial presentation. The diagnosis that resulted from applying the ICHD-3 and ICHD-3 beta diagnostic criteria was compared to the diagnosis made independently by the treating physicians performing the usual diagnostic work-up.

**Results:**

The new ICHD-3 diagnostic criteria for migraine with aura and migraine with typical aura display an excellent specificity (96 and 98% respectively), and are significantly more specific than the previous ICHD-3 beta classification system when it comes to diagnosing a first single attack (*probable* migraine with aura and *probable* migraine with typical aura).

**Conclusions:**

The ICHD-3 is a highly useful tool for the clinical neurologist in order to distinguish between a migraine with aura and a TIA, already at the first point of patient contact, such as in the emergency department or a TIA clinic.

## Background

A suspected transient ischemic attack (TIA) is a situation with particularly high uncertainty among emergency physicians [1]. A migraine with aura easily mimics a TIA, and regularly, patients with TIA are discharged as migraine with aura, since migraine with aura is the third most common stroke mimic, following seizures and psychiatric disorders [2].

The diagnosis of migraine with aura and migraine with typical aura has been systematized for the first time with the publication of the International Classification of Headache disorders (ICHD-1) [3] with updated criteria published as the second edition (ICHD-2) in 2004 [4] and most recently a third edition (ICHD-3) in 2018 [5]. One of the changes that was introduced from ICHD-2 via ICHD-3 beta to ICHD-3 was a change to the diagnostic criteria of migraine with aura (chapter 1.2) and migraine with typical aura (chapter 1.2.1), with the aim of being able to better differentiate migraines from transient ischemic attacks. Changes between the two most recent versions (ICHD-3 beta and ICHD-3) can be found in Additional file 1: Table S1.

The aim of our study was to assess, whether these changes result in a higher specificity when diagnosing migraine with aura and migraine with typical aura.

## Methods

### Study population

The study was conducted on a total of 128 patients who presented to the emergency room of the University Hospital of Lübeck, Germany between August 2016 and January 2017 and were referred for suspected transient ischemic attack. Reasons for exclusion were: previous stroke, subarachnoid heamorrhage, intracranial aneurysm, intracranial haemorrhage, brain tumor, any operation on the brain, multiple sclerosis, epilepsy, encephalitis, meningitis, dementia or memory problems, speech problems and other serious neurological or somatic disorder. Patients were recruited at the time of presentation to the emergency room interviewed by a member of the study team, usually still in the emergency room, and at the latest within 8 h of presentation. The interview took place independently from the standard care that patients received. A semi-structured interview was conducted by a junior doctor (CHG) or a senior medical student (SCK), both with a special interest in migraine and documented in a set format. Specifically, questions were asked focusing on the ICHD-3 diagnostic criteria of 1.2 Migraine with aura and 1.2.1 Migraine with typical aura, as well as those of the recently superseded ICHD-3 beta diagnostic criteria, in order to determine and compare the specificity of the old and new classification system. Additional medical background information such as the past medical history (including arterial hypertension, diabetes, atrial fibrillation, migraine), medication history and social history were collected. Family history of migraine was not recorded. We also calculated the body mass index (BMI) and ABCD^2^ score [6] of each subject.

The patients received standard care during the subsequent hospital stay (all 128 participants that were recruited to the study were independently admitted as an inpatient) from physicians independent from the study team and blinded to the collected data. The workup consisted of a DWI-MRI of the brain, extracranial and transcranial duplexsonography and a 24-h ECG. Using all the diagnostic information, a final diagnosis was performed by the treating senior neurologist at the time of discharge. This neurologist was also blinded to the results of the structured interview performed by the study team. The diagnosis of a TIA was made according to the AHA/ASA definition, i.e. patients with DWI positive lesions were classified as having had a stroke.

### Ethical considerations

The Medical Ethics Committee of the University of Lübeck had approved this study prior to its start. All participants were informed of the purpose of the study. Written informed consent was obtained from all participants.

### Statistical analysis

We tested the specificity of:

- the diagnostic criteria for migraine with aura and migraine with typical aura of the ICHD-3 [5] and
- of the ICHD-3 beta [7]

in our 128 patients with suspected TIA.

Specificity was calculated as the number of true negatives relative to all subjects actually being negative for the specific analysis (i.e. true negatives + false positives). Statistical calculations were made using McNemar’s Chi-Square Test.

## Results

A total of 158 patients were considered for enrolment, 30 of which did not meet the inclusion criteria (27 were excluded due to a previous stroke and 3 due to pre-existing dementia). The mean age of the 128 enrolled patients (63 women, 49.2%) was 68 years (SD 13.3 years). One hundred eight of the 128 patients (84.4%) were self-referrals and arrived by ambulance (*n* = 58, 45.3%) or by private transportation (*n* = 50, 39.1%). Twenty-eight patients (21.9%) were referred from their primary care doctor, all of whom also arrived by ambulance.

Symptom duration of patients (including those that went on to have symptoms longer than 24 h and thus were discharged with a diagnosis other than TIA, most usually a stroke) was a median of 47.5 min (interquartile range: 10–120 min). The symptom duration of those patients, that were in the end discharged with the diagnosis of a TIA (*n* = 78) was a median of 30 min (interquartile range: 5–112.5 min).

Of the 78 TIA patients, the most frequently experienced TIA symptoms were sensory deficits (32.1%), motor deficits (25.6%) and brainstem symptoms (24.4%). 55 (70.5%) patients had only one symptom in isolation, 19 (24.4%) showed 2 symptoms, 3 (3.8%) patients had 3 symptoms and 1 patient (1.3%) suffered from 4 symptoms. A detailed breakdown of frequency of symptom type and individual duration can be found in Additional file 1: Table S7.

Four out of the 128 patients (3.125%) reported a prior migraine diagnosis. Additional sociodemographic data of all patients and duration profile of each type of symptoms are documented in the Additional file 1.

### Diagnosis of discharge

Seventy-eight patients (60.9%) referred under the suspicion of a TIA were actually classified as having suffered a TIA, 31 patients (24.2%) an ischemic cerebral infarct, 4 patients (3.1%) a migraine with aura, 3 patients (2.3%) an epilepsy, 2 patients (1.6%) a somatoform disorder, and 1 patient each (0.8%) a number of 10 further diagnoses, each diagnosed once (abducens nerve palsy, syncope, benign paroxysmal positional vertigo, Ménière’s disease, Parkinson’s disease, inflammatory CNS disease, transient global amnesia, cancer of unknown primary origin syndrome, keratitis and hypertensive encephalopathy).

The average BMI was 27.8 kg/m^2^ and did not differ significantly between men and women and patients who were discharged with a TIA versus those who were not (Additional file 1: Tables S8 and S9).

The average ABCD^2^ score of all TIA patients was 3.21 (Additional file 1: Tables S10 and S11). When looking at all 20 patients that neither received the diagnosis of a TIA nor that of an ischemic cerebral infarct, we found that their ABCD^2^ score was significantly lower at 2.15 (*p* < 0.001).

### Specificity of ICHD-3 and ICHD-3 beta diagnostic criteria for migraine with aura and migraine with typical aura

**Fig. 1:**
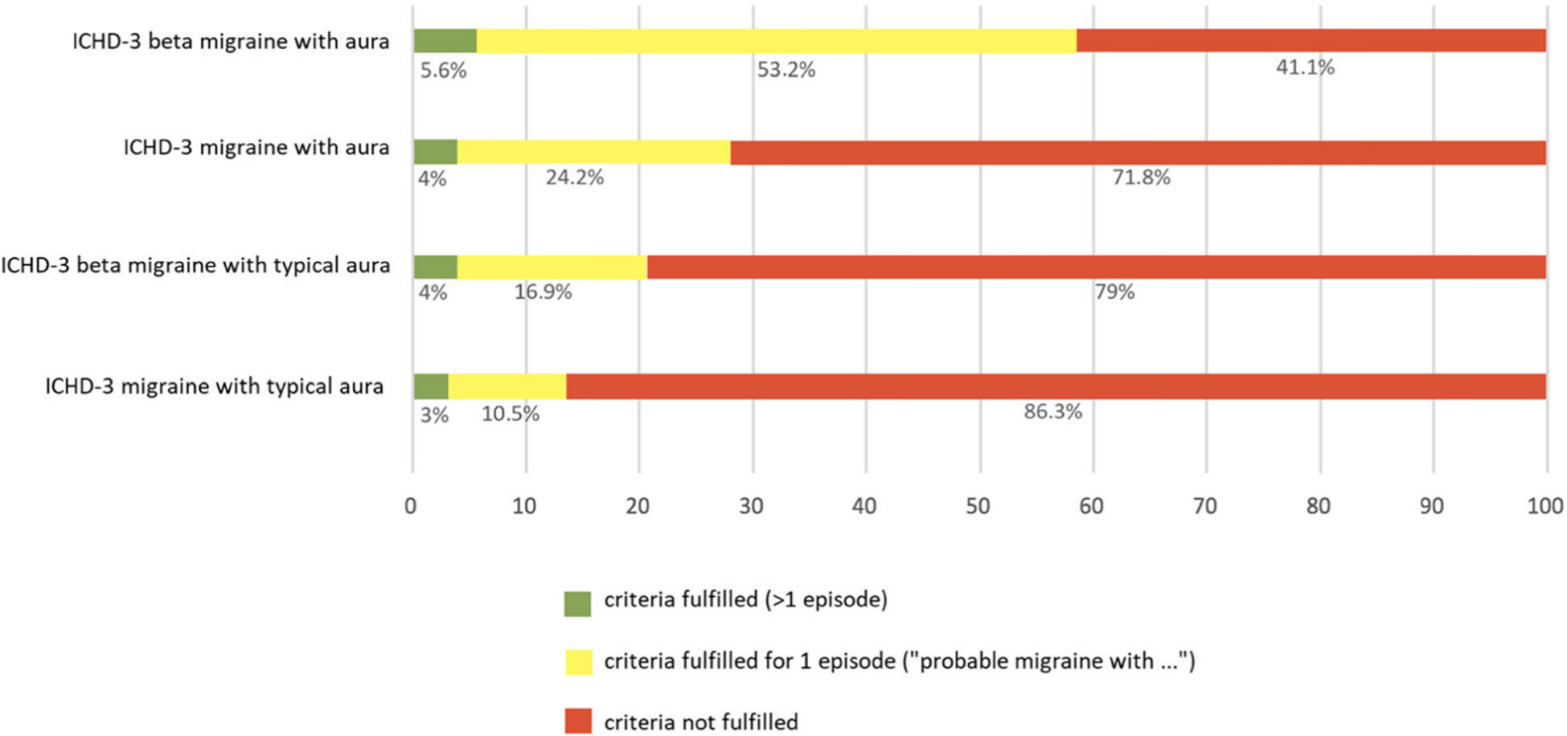
Percentage of ICHD-3 beta and ICHD-3 diagnoses being positive or negative (where stated) in the 124 patients not discharged with a diagnosis of a migraine

Applying the ICHD-3 and ICHD-3 beta diagnostic criteria for migraine with aura and migraine with typical aura to the 124 patients not discharged with a diagnosis of a migraine, we found that either set of criteria had a low rate of false positive diagnosis of both migraine with aura (4% in ICHD-3 and 5.6% in ICHD-3 beta, Fig. 1) and migraine with typical aura (3% in ICHD-3 and 4% in ICHD-3 beta, Fig. 1) resulting in a specificity between 94% and 97%, Table 1.

**Table 1:**
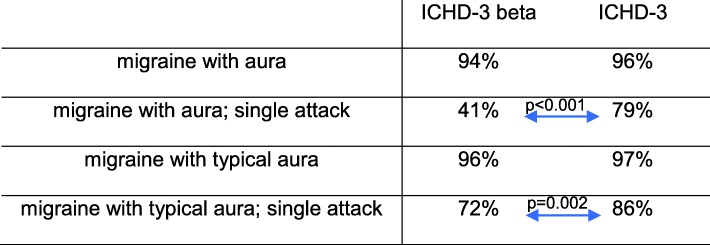
Specificity calculation for the ICHD-3 and ICHD-3 beta diagnoses (McNemar’s Chi-Square Test for statistical calculations)

If we however look at the diagnosis of a single migraine attack (*probable* migraine with aura, whereby only one attack has occurred), the ICHD-3 beta results in a false-positive rate of 53.2% of our TIA patients (specificity of only 41%), while the ICHD-3 has a lower false-positive rate of only 24.2% (79% specificity). The specificity of the ICHD-3 for a single attack of migraine with aura was significantly greater than that of the ICHD-3 beta (*p* < 0.001, McNemar’s Chi-Square Test).

A similar pattern holds true for the diagnostic criteria of a single attack of migraine with *typical* aura. Here, the ICHD-3 beta has a false-positive rate of 16.9% of our patient population (specificity of 72%), while the ICHD-3 has a lower rate of 10.5% (specificity of 86%). The specificity of the ICHD-3 for single attack migraine with typical aura is thus significantly greater than that of the ICHD-3 beta (*p* = 0.002, McNemar’s Chi-Square Test). Given the fact that all 4 patients discharged with migraine were correctly classified by both the ICHD-3 and ICHD-3 beta criteria, and thus had a sensitivity of 100%, the negative likelihood ratio is 0 in all cases. The positive likelihood ratios can be seen in Table 2.

**Table 2:** Positive likelihood ratios for the ICHD-3 and ICHD-3 beta diagnoses (the negative likelihood ratios were 0 throughout)

	ICHD-3 beta	ICHD-3
migraine with aura	17.7	24.8
migraine with aura; single attack	1.7	4.8
migraine with typical aura	24.8	31.0
migraine with typical aura; single attack	3.5	7.3

### Characterising patients with a TIA diagnosis, with a migraine diagnosis and patients who were false positively diagnosed with a migraine according to ICHD-3

In an attempt to find differentiating characteristics useful for clinical practice, we performed a sub-group analysis of the following three groups:

- Group A: patients who received a discharge diagnosis of a TIA (*n* = 78)
- Group B: patients who received a discharge diagnosis of a migraine (*n* = 4)
- Group C: patients who fulfilled ICHD-3 diagnostic criteria for migraine with aura or typical aura (one or more episodes), but who received a discharge diagnosis of a TIA (i.e. migraine with aura false positives) (*n* = 17)

The data are shown in Table 3. Patients with migraine were significantly younger than patients with a TIA (*p* = 0.0042) and also than their false positive counterparts of Group C (*p* = 0.0374), meaning that younger age is an important positive predictor of a true positive migraine diagnosis. The other characteristics shown did not differ significantly between the three groups.

**Table 3:** Sub-group analysis

	Group A patients diagnosed with TIA; *n* = 78	Group B patients diagnosed with migraine with aura; *n* = 4	Group C patients fulfilling ICHD-3 criteria for migraine with aura for one or more attacks, but receiving a final diagnosis of a TIA (“migraine with aura false positives”); *n* = 17
Age (± standard deviation)	70.9 (± 12.7) years	51.5 ± 12.1 years	69.9 ± 14.5 years
Number of female patients	38 (48.7%)	3 (75%)	11 (64.7%)
Number of patients with arterial hypertension	52 (66.6%)	2 (50%)	11 (64.7%)
Number of patients with hypercholesterinemia	45 (57.7%)	3 (75%)	9 (52.9%)
Number of patients with atrial fibrillation (including new diagnoses)	15 (19.2%), 1 newly diagnosed	1 (25%), none newly diagnosed	2 (11.8%), none newly diagnosed
Number of patients with carotid artery plaque or stenosis	18 (23.1%)	1 (25%)	7 (41.2%)
Number of patients with prior stroke found in MRI	9 (11.5%)	0	3 (17.6%)

## Discussion

Distinguishing between a TIA and a migraine with aura is not always straightforward and mistakes are very harmful: Misdiagnosing a migraine patient with a TIA renders him or her to an unnecessary expensive diagnostic work-up as well as lifelong antiplatelet and lipid-lowering therapy, while misdiagnosing a TIA as a migraine with aura may result in an avoidable stroke [8]. Monetary incentives, whereby the diagnosis of a TIA is reimbursed more than a migraine with aura, may also introduce conflicts of interest in the healthcare setting.

In order to make the diagnosis of a migraine with aura and migraine with typical aura, a patient needs to have a history of at least 2 episodes fulfilling the criteria. If the patient presents with a first episode, it becomes even more challenging to decide whether this is a migraine attack or a TIA (a previous similar episode makes the diagnosis of a migraine far more likely). Hence it is these patients, where a precise (i.e. specific) diagnostic classification system is most important in clinical practice.

Arriving at any medical diagnosis involves the weighing up of evidence. Usually, a greater or lesser degree of diagnostic uncertainty will remain. The diagnostic “gold standard” that we used in our study was the opinion of a senior neurologist at a German tertiary hospital, which in our opinion is the best approximation to the correct diagnosis. Patients with symptoms of a TIA as well as a migraine however often initially present to doctors less experienced in the treatment of neurological disorders, such as their general practitioner or an ophthalmologist in case of visual symptoms. Here, even more than for the tertiary sector, a clinically-oriented classification system is important to guide further diagnostic and therapeutic work-up.

There are a number of limitations of our study. The assessors performing the survey were not blinded to the study design. However, the final diagnosis was performed independently by a senior neurologist at time of discharge (who was not aware of the study being performed). This meant that any potential bias was minimized, as often the final diagnosis differed from the suspected diagnosis at time of presentation when the survey was carried out.

Additionally, the low prevalence of migraine with aura (3.1% or 4 patients) in our study population could lead to spurious specificity measurements. A reason for the low prevalence is likely to be the fact that many patients with a clear migraine disorder were filtered out prior to admission to this tertiary centre, after being assessed by an outpatient doctor or by the ambulance crew. All 4 patients were correctly classified as having had a migraine with aura by both the ICHD-3 beta and ICHD-3, so the increased specificity of the ICHD-3 does not appear to come at the price of a worsened sensitivity.

Our diagnostic gold standard of the assessment of a single senior neurologist at the time of discharge may be problematic, as even senior neurologists can misdiagnose TIA and headache. All of the patients included in our study however were treated by at least 2 consultant neurologists during the course of their admission (one at the stroke unit and one at the general ward) and a diagnostic evaluation was also performed by the head of department at his weekly ward round. Hence we feel our applied gold standard is approximating the true diagnosis as good as it can.

Our study shows that the new ICHD-3 diagnostic criteria for migraine with aura and migraine with typical aura display a very good specificity, and are significantly more specific than the previous ICHD-3 beta classification system when it comes to diagnosing a single attack of migraine with aura and with typical aura. Thus, the ICHD-3 presents a useful tool for the clinical neurologist to distinguish between a migraine with aura and a TIA, already at the first point of patient contact, such as in the emergency department or a TIA clinic.

## Conclusions

- Migraine with aura is difficult to distinguish from a transient ischemic attack, mistakes are harmful to both patient and society.
- The new ICHD-.3 diagnostic criteria for migraine with aura and migraine with typical aura display an excellent specificity, particularly for first, single attacks.
- The ICHD-3 is a highly useful tool for the clinical neurologist in order to distinguish between a migraine with aura and a TIA, already at the first point of patient contact, such as in the emergency department or a TIA clinic.

## Supplementary information

**Additional file 1: Table S1.** ICHD criteria for the diagnosis of migraine with aura. **Table S2.** ICHD criteria for migraine with typical aura. **Table S3.** Distribution of patients referred under the suspicion of TIA (*n* = 128) by age and sex. **Table S4.** Distribution of patients discharged with TIA diagnosis by age and sex (*n* = 78) by age and sex. **Table S5.** Sociodemographic data of patients referred under the suspicion of TIA (*n* = 128). **Table S6.** Sociodemographic data of patients discharged with TIA diagnosis (*n* = 78). **Table S7.** Duration and type of symptoms in patients discharged with TIA (*n* = 78). **Table S8.** BMI of patients referred under the suspicion of TIA (*n* = 128). **Table S9.** BMI of patients discharged with TIA diagnosis (*n* = 78). **Table S10.** ABCD2 score of patients referred under the suspicion of TIA (*n* = 128), average score: 3.35. **Table S11.** ABCD2 score of patients discharged with TIA diagnosis (*n* = 78), average score: 3.21.

## Data Availability

The datasets during and/or analysed during the current study available from the corresponding author on reasonable request.

## References

[1] Eagles D, Stiell IG, Clement CM, Brehaut J, Kelly AM, Mason S (2008). International survey of emergency physicians’ priorities for clinical decision rules. Acad Emerg Med.

[2] Terrin A, Toldo G, Ermani M, Mainardi F, Maggioni F (2018). When migraine mimics stroke: a systematic review. Cephalalgia.

[3] IHS-Classification-Committee (1988). Classification and diagnostic criteria for headache disorders, cranial neuralgias and facial pain. Cephalalgia.

[4] IHS-Classification-Committee (2004). The international classification of headache disorders. Cephalalgia.

[5] Olesen J (2018). Headache Classification Committee of the International Headache Society (IHS) the international classification of headache disorders, asbtracts. Cephalalgia.

[6] Rothwell P, Giles M, Flossmann E, Lovelock C, Redgrave J, Warlow C (2005). A simple score (ABCD) to identify individuals at high early risk of stroke after transient ischaemic attack. Lancet.

[7] Society HCCotIH (2013). The international classification of headache disorders, (beta version). Cephalalgia.

[8] Kuruvilla A, Bhattacharya P, Rajamani K, Chaturvedi S (2011). Factors associated with misdiagnosis of acute stroke in young adults. J Stroke Cerebrovasc Dis.

