# Supplementary material for: ICHD-3 is significantly more specific than ICHD-3 beta for diagnosis of migraine with aura and with typical aura
Source: J Headache Pain. 2020 Jan 7;21(1):2. doi: 10.1186/s10194-019-1072-2 (PMC6947981; doi:10.1186/s10194-019-1072-2)
Supplement: Supplementary file 1 — Additional file 1: Table S1. ICHD criteria for the diagnosis of migraine with aura. Table S2. ICHD criteria for migraine with typical aura. Table S3. Distribution of patients referred under the suspicion of TIA (n = 128) by age and sex. Table S4. Distribution of patients discharged with TIA diagnosis by age and sex (n = 78) by age and sex. Table S5. Sociodemographic data of patients referred under the suspicion of TIA (n = 128). Table S6. Sociodemographic data of patients discharged with TIA diagnosis (n = 78). Table S7. Duration and type of symptoms in patients discharged with TIA (n = 78). Table S8. BMI of patients referred under the suspicion of TIA (n = 128). Table S9. BMI of patients discharged with TIA diagnosis (n = 78). Table S10. ABCD2 score of patients referred under the suspicion of TIA (n = 128), average score: 3.35. Table S11. ABCD2 score of patients discharged with TIA diagnosis (n = 78), average score: 3.21. [file 10194_2019_1072_MOESM1_ESM.docx]

**Additional file 1**

**Table S1** ICHD criteria for the diagnosis of migraine with aura

| ICHD-3 beta 1.2 Migraine with aura | ICHD-3 1.2 Migraine with aura |
| --- | --- |
| A. At least two attacks fulfilling criteria B and C  B. One or more of the following fully reversible aura symptoms:  1. Visual  2. Sensory  3. Speech and/or language  4. Motor  5. Brainstem  6. Retinal  C. At least two of the following four characteristics:  1. At least one aura symptom spreads gradually over 5 minutes, and/or two or more symptoms occur in succession  2. Each individual aura symptom lasts 5–60 minutes  3. At least one aura symptom is unilateral  4. The aura is accompanied, or followed within 60 minutes, by headache  D. Not better accounted for by another ICHD-3 diagnosis, and transient ischaemic attack has been excluded. | A. At least two attacks fulfilling criteria B and C  B. One or more of the following fully reversible aura symptoms:  1. Visual  2. Sensory  3. Speech and/or language  4. Motor  5. Brainstem  6. Retinal  C. At least three of the following six characteristics:  1. At least one aura symptom spreads gradually over 5 minutes  2. Two or more aura symptoms occur in succession  3. Each individual aura symptom lasts 5–60 min  4. At least one aura symptom is unilateral  5. At least one aura symptom is positive  6. The aura is accompanied, or followed within 60 minutes, by headache  D. Not better accounted for by another ICHD-3 diagnosis. |

**Table S2** ICHD criteria for migraine with typical aura

| ICHD-3 beta 1.2.1 Migraine with typical aura | ICHD-3 1.2.1 Migraine with typical aura |
| --- | --- |
| A. At least two attacks fulfilling criteria B and C  B. Aura consisting of visual, sensory and or speech/language symptoms, each fully reversible, but no motor, brainstem or retinal symptoms  C. At least two of the following four characteristics:  1. At least one aura symptom spreads gradually over 5 minutes, and/or two or more symptoms occur in succession  2. Each individual aura symptom lasts 5–60 minutes  3. At least one aura symptom is unilateral  4. The aura is accompanied, or followed within 60 minutes, by headache  D. Not better accounted for by another ICHD-3 diagnosis, and transient ischaemic attack has been excluded. | A. At least two attacks fulfilling criteria B and C  B. Aura consisting of visual, sensory and or speech/language symptoms, each fully reversible, but no motor, brainstem or retinal symptoms  C. At least three of the following six characteristics:  1. At least one aura symptom spreads gradually over 5 minutes  2. Two or more aura symptoms occur in succession  3. Each individual aura symptom lasts 5–60 minutes  4. At least one aura symptom is unilateral  5. At least one aura symptom is positive  6. The aura is accompanied, or followed within 60 minutes, by headache  D. Not better accounted for by another ICHD-3 diagnosis. |

**Table S3.** Distribution of patients referred under the suspicion of TIA (n = 128) by age and sex

| Sex and age interval | 36-45 | 46-55 | 56-65 | 66-75 | 76-90 |
| --- | --- | --- | --- | --- | --- |
| men (n= 65) | 5 (7.7%) | 10 (15.4%) | 8 (12.3%) | 23 (35.4%) | 19 (29.2%) |
| women (n= 63) | 5 (7.9%) | 9 (14.3%) | 8 (12.7%) | 18 (28.6%) | 23 (36.5%) |
| All (n= 128) | 10 (7.8%) | 19 (14.8%) | 16 (12.5%) | 41 (32.0%) | 42 (32.8%) |

**Table S4.** Distribution of patients discharged with TIA diagnosis by age and sex (n = 78) by age and sex

| Sex and age interval | 36-45 | 46-55 | 56-65 | 66-75 | 76-90 |
| --- | --- | --- | --- | --- | --- |
| men (n= 40) | 3 (7.5%) | 4 (10.0%) | 5 (12.5%) | 14 (35.0%) | 14 (35.0%) |
| women (n= 38) | 1 (2.6%) | 3 (7.9%) | 5 (13.2%) | 9 (23.7%) | 20 (52.6%) |
| All (n= 78) | 4 (5.1%) | 7 (9.0%) | 10 (12.8%) | 23 (29.5%) | 34 (43.6%) |

**Table S5**. Sociodemographic data of patients referred under the suspicion of TIA (n = 128)

| Sociodemographic characteristics | Males (n = 65) | Females (n = 63) | All (n= 128) |
| --- | --- | --- | --- |
| Mean age (years) | 67.5 | 68.7 | 68.0 |
| Age interval | 42-91 | 37-92 | 37-92 |
| Education: general secondary school | 31 (47.7 %) | 33 (52.4 %) | 64 (50.0 %) |
| Education: intermediate secondary school | 19 (29.2 %) | 18 (28.6 %) | 37 (28.9 %) |
| Education: grammar school | 4 (6.2 %) | 6 (9.5 %) | 10 (7.8 %) |
| Education: university | 11 (16.9 %) | 3 (4.8 %) | 14 (10.9 %) |
| Employment: Student | 0 (0.0 %) | 0 (0.0 %) | 0 (0.0 %) |
| Employment: Employed | 20 (30.8 %) | 20 (31.7 %) | 40 (31.3 %) |
| Employment: Entrepreneur | 9 (13.8 %) | 0 (0.0 %) | 9 (7.0 %) |
| Employment: Unemployed | 0 (0.0 %) | 1 (1.6 %) | 1 (0.8 %) |
| Employment: Pensioner | 36 (55.4 %) | 42 (66.7 %) | 78 (60.9 %) |

**Table S6**. Sociodemographic data of patients discharged with TIA diagnosis (n = 78)

| Sociodemographic characteristics | Men (n = 40) | Women (n = 38) | All (n= 78) |
| --- | --- | --- | --- |
| Mean age (years) | 67.5 | 73.2 | 70.9 |
| Age interval | 42-91 | 37-92 | 37-92 |
| Education: general secondary school | 17 (42.5 %) | 23 (60.5 %) | 40 (51.3 %) |
| Education: intermediate secondary school | 15 (37.5 %) | 8 (21.1 %) | 23 (29.5 %) |
| Education: grammar school | 1 (2.5 %) | 5 (13.2 %) | 6 (7.7 %) |
| Education: university | 7 (17.5 %) | 2 (5.3 %) | 9 (11.5 %) |
| Employment: Student | 0 (0.0 %) | 0 (0.0 %) | 0 (0.0 %) |
| Employment: Employed | 11 (27.5 %) | 9 (23.7 %) | 20 (25.6 %) |
| Employment: Entrepreneur | 4 (10.0 %) | 0 (0.0 %) | 4 (5.1 %) |
| Employment: Unemployed | 0 (0.0 %) | 1 (2.6 %) | 1 (1.3 %) |
| Employment: Pensioner | 25 (62.5 %) | 28 (73.7 %) | 53 (67.9 %) |

**Table S7**. Duration and type of symptoms in patients discharged with TIA (n = 78)

|  | Duration of TIA symptoms | | | | All patients |
| --- | --- | --- | --- | --- | --- |
| Symptoms of TIA | 2-5mins | 16mins-1h | 1-3h | 3-24h |  |
| Sensory deficits | 9 (11.5 %) | 5 (6.4 %) | 4 (5.1 %) | 7 (9.0 %) | 25 (32.1 %) |
| Motor deficits | 6 (7.7 %) | 6 (7.7 %) | 2 (2.6 %) | 6 (7.7 %) | 20 (25.6 %) |
| Aphasia | 4 (5.1 %) | 4 (5.1 %) | 1 (1.3 %) | 2 (2.6 %) | 11 (14.1 %) |
| Monocular (retinal) deficits | 11 (14.1 %) | 0 (0.0 %) | 0 (0.0 %) | 0 (0.0 %) | 11 (14.1 %) |
| Brainstem symptoms | 8 (10.3 %) | 9 (11.5 %) | 0 (0.0 %) | 2 (2.6 %) | 19 (24.4 %) |
| Dysarthria | 1 (1.3 %) | 2 (2.6 %) | 1 (1.3 %) | 1 (1.3 %) | 5 (6.4 %) |
| Binocular (central) deficits | 5 (6.4 %) | 1 (1.3 %) | 0 (0.0 %) | 2 (2.6 %) | 8 (10.3 %) |

**Table S8**. BMI of patients referred under the suspicion of TIA (n = 128)

|  | Males (n = 65) | Females (n = 63) | All (n = 128) |
| --- | --- | --- | --- |
| BMI average | 27.3 kg/m^2^ | 28.3 kg/m^2^ | 27.8 kg/m^2^ |

**Table S9**. BMI of patients discharged with TIA diagnosis (n = 78)

|  | Males (n = 40) | Females (n = 38) | All (n = 78) |
| --- | --- | --- | --- |
| BMI average | 26.1 kg/m^2^ | 27.6 kg/m^2^ | 28.8 kg/m^2^ |

**Table S10**. ABCD2 score of patients referred under the suspicion of TIA (n = 128), average score: 3.35

| ABCD2 score | Males (n = 65) | Females (n = 63) | All (n = 128) |
| --- | --- | --- | --- |
| ABCD2 = 0 | 3 (2.4 %) | 2 (1.6%) | 5 (3.9%) |
| ABCD2 = 1 | 7 (5.5 %) | 6 (4.7%) | 13 (10.2%) |
| ABCD2 = 2 | 11 (8.7 %) | 14 (11.0%) | 25 (19.7%) |
| ABCD2 = 3 | 11 (8.7 %) | 11 (8.7%) | 22 (17.3%) |
| ABCD2 = 4 | 16 (12.6 %) | 11 (8.7%) | 27 (21.3%) |
| ABCD2 = 5 | 10 (7.9 %) | 12 (9.4%) | 22 (17.3%) |
| ABCD2 = 6 | 6 (4.7 %) | 6 (4.7%) | 12 (9.4%) |
| ABCD2 = 7 | 1 (0.8 %) | 0 (0.0%) | 1 (0.8%) |

**Table S11**. ABCD2 score of patients discharged with TIA diagnosis (n = 78), average score: 3.21

| ABCD2 score | Males (n = 40) | Females (n = 38) | All |
| --- | --- | --- | --- |
| ABCD2 = 0 | 2 (1.6 %) | 1 (0.8 %) | 3 (2.4 %) |
| ABCD2 = 1 | 5 (3.9 %) | 3 (2.4 %) | 8 (6.3 %) |
| ABCD2 = 2 | 8 (6.3 %) | 9 (7.1 %) | 17 (13.4 %) |
| ABCD2 = 3 | 9 (7.1 %) | 8 (6.3 %) | 17 (13.4 %) |
| ABCD2 = 4 | 7 (5.5 %) | 6 (4.7 %) | 13 (10.2 %) |
| ABCD2 = 5 | 5 (3.9 %) | 8 (6.3 %) | 13 (10.2 %) |
| ABCD2 = 6 | 3 (2.4 %) | 2 (1.6 %) | 5 (3.9 %) |
| ABCD2 = 7 | 1 (0.8 %) | 0 (0.0 %) | 1 (0.8 %) |
